# Seth Green’s Spider Story

**Published:** 1881-08

**Authors:** 


### SETH GREEN'S SPIDER STORY.

If you anchor a pole in a body of water and put a spider upon it he will exhibit marvelous intelligence by his plans to escape. At first he will spin a web several inches long and hang to one end while he allows the other to float off in the wind, in the hope that it will strike some object. Of course this plan proves a failure, but the spider is not discouraged. He waits until the wind changes, and then sends another silken bridge floating off in another direction. Another failure is followed by several other similar attempts, until all the points of the compass have been tried. But neither the resources nor the reasoning powers of the spider have been exhausted. He climbs to the top of the pole and energetically goes to work to construct a silken balloon. He has no hot air with which to inflate it. but he has the power of making it buoyant. When he gets his balloon finished he does not go off upon the mere supposition that it will carry him, as men often do, but he fastens it to a guy rope, the other end of which he attaches to the island pole upon which he is a prisoner. He then gets into his aerial vehicle, while it is made fast, and tests it to see whether its dimensions are capable of the work of bearing him away. He often finds that he has made it too small, in which case he hauls it down, takes it all apart, and constructs it on a larger and better plan. A spider has been seen to make three different balloons before he became satisfied with his experiment. Then he will get in, snap the guy rope, and sail away to land as gracefully and as supremely independent of his surroundings as could well be imagined.

